# Reliability of the interRAI suite of assessment instruments: a 12-country study of an integrated health information system

**DOI:** 10.1186/1472-6963-8-277

**Published:** 2008-12-30

**Authors:** John P Hirdes, Gunnar Ljunggren, John N Morris, Dinnus HM Frijters, Harriet Finne Soveri, Len Gray, Magnus Björkgren, Reudi Gilgen

**Affiliations:** 1 University of Waterloo, 200 University Ave W, Waterloo, ON N2L 3G1, Canada; 2 Homewood Research Institute, Guelph, Canada; 3 Stockholm County Council, Box 17533, S-11891, Stockholm, Sweden; 4 Hebrew Senior Life, 1200 Centre St, Boston, MA 02131, USA; 5 PRISMANT, Box 14006, Papendorpseweg 65, 3528 BJ Utrecht, The Netherlands; 6 STAKES, Lintulahdenkuja 4, Box 220, FIN-00531, Helsinki, Finland; 7 University of Queensland, Academic Unit in Geriatric Medicine, Princess Alexandra Hospital, Ipswich Road, Woolloongabba, Brisbane, Australia; 8 Chydenius Institute, Pitkänsillankatu 1-3, 67100 Kokkola, Finland; 9 Stadtspital Waid Zürich, Klinik für Akutgeriatrie, Tièchestraße 99, CH-8037, Zürich, Switzerland

## Abstract

**Background:**

A multi-domain suite of instruments has been developed by the interRAI research collaborative to support assessment and care planning in mental health, aged care and disability services. Each assessment instrument comprises items common to other instruments and specialized items exclusive to that instrument. This study examined the reliability of the items from five instruments supporting home care, long term care, mental health, palliative care and post-acute care.

**Methods:**

Paired assessments on 783 individuals across 12 nations were completed within 72 hours of each other by trained assessors who were blinded to the others' assessment. Reliability was tested using weighted kappa coefficients.

**Results:**

The overall kappa mean value for 161 items which are common to 2 or more instruments was 0.75. The kappa mean value for specialized items varied among instruments from 0.63 to 0.73. Over 60% of items scored greater than 0.70.

**Conclusion:**

The vast majority of items exceeded standard cut-offs for acceptable reliability, with only modest variation among instruments. The overall performance of these instruments showed that the interRAI suite has substantial reliability according to conventional cut-offs for interpreting the kappa statistic. The results indicate that interRAI items retain reliability when used across care settings, paving the way for cross domain application of the instruments as part of an integrated health information system.

## Background

Population aging and the increased burden of disability in middle and high income nations pose unique challenges to health care systems. The lives of frail elderly individuals and persons with disability are affected by complex interactions of physical, social, medical and environmental factors that necessitate multidisciplinary approaches to care. Services tend to be provided by a variety of health and social service agencies including both community and facility-based settings. For example, persons who are experiencing cognitive loss or decline of functional ability may receive support from home care agencies, supportive housing, rehabilitation services, or nursing homes. Similarly, persons with mental health problems may receive psychiatric services in primary care, community mental health programs, mental health group homes, or in-patient psychiatric units of hospitals. At the end of life, palliative care may be provided by community-based agencies or by residential hospices, but periodic contact with acute hospitals is also not uncommon. For each of these populations, health and social services are intended to be provided through an integrated system of care rather than through a singular organization.

The need to receive support from multiple service agencies has important implications for persons with complex care needs. At the individual level, there may be a risk of discontinuity of care if information systems are not compatible or if clinically relevant information is not shared between agencies. This may mean that needs are not identified[1] when transitions are made between service providers, longitudinal change in functional status may go undetected as the person moves between service settings, or care plans are not followed through when the person receives care from another sector. The lack of coordination of information gathering can result in duplication of effort, increased assessment burden, and frustration among care recipients and their support network. For these reasons, there is a clear need for an integrated, multi-sectoral approach to assessment for persons with complex care needs.

The interRAI family of assessment instruments  was designed to be used with a variety of vulnerable populations [2,3]. The first interRAI instrument was the Resident Assessment Instrument (RAI), developed in the United States in response to the Omnibus Reconciliation Act of 1987 [4]. The interRAI network was established initially based on the international collaborative efforts of clinicians and researchers to apply the RAI to nursing home residents in other countries [5]. By 1996, interRAI released the RAI-Home Care with the aim of establishing a compatible assessment approach in community based care settings that served populations at risk of nursing home placement or required post-acute or long term home care services [6-8]. The RAI-Mental Health instrument [9,10] was the first interRAI instrument designed to be used with a general adult population in psychiatric hospital settings including, but not limited to, geriatric psychiatry. Other interRAI instruments developed in the 1990's include the RAI-Acute Care [11,12], RAI-Post Acute Care [13], and RAI-Palliative Care [14].

The development of all these assessment instruments was guided by the design principles for the original RAI. The assessments were intended to use all sources of information available. Judgments were to be based on observable traits, have operational definitions and coding instructions that specified inclusion and exclusion criteria, and use clearly delimited time frames for observations anchored to a specific assessment reference date. In addition, each of these instruments was intended to support applications for multiple audiences including care planning, outcome measurement, quality improvement, and resource allocation. To this end, efforts were made to retain the capacity to derive or extend existing outcome measures (e.g., scales related to cognition, ADL, pain, depression, behaviour) and decision support algorithms (e.g., case mix algorithms, quality indicators).

The initial set of RAI instruments was developed in a serial process. For new instruments, this meant that lessons learned from the use of earlier instruments were taken into account in the development of subsequent instruments. However, the need to refine older instruments became apparent as innovations were identified. Also, it was recognized that the family of instruments should be refined in parallel, treating the collective set of instruments as an integrated system rather than as complementary, but independent assessments for specialized care settings.

In the year 2000, interRAI launched a multinational effort to update the entire family of RAI instruments and to develop new instruments for sectors not yet addressed by the existing instruments. The result of this effort is an integrated suite of instruments providing compatible assessment approaches for nursing homes, home care, acute care, post acute care, palliative care, assisted living, supportive housing, services for persons with intellectual disabilities, community mental health, emergency psychiatry, and inpatient psychiatry. The initial focus of the redevelopment effort for the interRAI suite of assessment instruments was to identify a common core set of about 70 items that would be present in all instruments, with exceptions permitted only for specialized settings where prevalence rates for the item will be negligible (e.g., pressure ulcers in in-patient psychiatry). Examples include items such as cognitive skills for decision making, activities of daily living (e.g., personal hygiene, toilet use, eating), mood (e.g., negative statements, persistent anger, crying/tearfulness), behaviour problems (e.g., verbal abuse, resisting care), falls, and health symptoms (e.g., pain frequency and intensity, fatigue). The next step was to identify over 100 optional items that would appear in many, but not all, instruments. These items were expected to be relevant to several service settings, but not pervasive enough in all service settings to warrant inclusion in the list of core items. Examples include long-term memory, situational memory, hearing aid use, family/close friends feeling overwhelmed by the person's illness, instrumental activities of daily living (e.g., meal preparation, financial management, phone use), stamina, additional health conditions (e.g., extrapyramidal symptoms, abnormal thought processes, delusions), medication adherence, and preventive interventions and screening (e.g., influenza vaccination, breast screening). Finally, specialized items that would only appear in specific instruments were also identified. For example, the in-patient psychiatry instrument has 170 unique measures (e.g., number of lifetime psychiatric admissions, command hallucinations, suicidality, illicit drug use, police intervention for criminal behaviour, history of sexual violence or assault as perpetrator, problem gambling) whose prevalences would be too low to warrant their use in non-mental health settings.

Once the initial item set was identified for the interRAI instruments, a 12-country effort was launched to evaluate the psychometric properties of the instruments in different health care settings. The present paper reports on the results of that cross-national effort with a particular emphasis on inter-rater reliability. There have been several studies of the reliability and validity of the early versions of interRAI instruments for nursing homes [15-20], home care[6,7,21], mental health [10], acute care [11], and palliative care [14]. The general trend of these studies has been to show improved reliability over time with newer versions of these instruments, and they provided consistent evidence of good psychometric properties across populations and service settings. The multinational effort described here was launched in 2005 and included five instruments from the new suite designed for use in the following care settings: nursing homes, home care, rehabilitation, palliative care, and in-patient psychiatry Results for the new interRAI Acute Care [22] and interRAI Intellectual Disability [23] have been reported elsewhere. Reliability and validity results for new instruments in the suite (e.g., interRAI Community Mental Health) will be reported in future publications.

## Methods

### Study Participants

interRAI Fellows from 12 countries (Australia, Canada, Czech Republic, France, Iceland, Italy, Japan, South Korea, Netherlands, Norway, Spain, and United States) volunteered to test one or more of five instruments available by 2005 for long-term care facilities, home care, palliative care, post-acute care, and mental health. Individual researchers selected instruments based on the availability of pilot sites in their countries, which was often dependent on patterns of use of the earlier versions of the instruments. For each instrument, interRAI created a detailed item by item instruction manual, with item definitions, process instructions, and examples. Field staff members were trained to do the assessments following this instructional set. These trained clinicians then completed dual assessments for 783 individuals. As shown in Table 1, the most widely tested instrument was the LTCF (8 countries, 31% of assessments) and the least common was MH (1 country, 11% of assessments). The largest number of assessments came from Canada (147 pairs), the United States (141 pairs), and Iceland (80 pairs), and the fewest were obtained from Spain (29 pairs) and Japan (28 pairs).

**Table 1:** Reliability samples by country and interRAI instrument

**Country**	**interRAI Assessment Instrument**						**Number of instruments evaluated**
	Long-Term Care Facility (LTCF)	Home Care (HC)	Palliative Care (PC)	Post-Acute Care (PAC)	Mental Health (MH)	All Instruments	
Australia		18		26		44	2
Canada	58				89	147	2
Czech republic		30	30			60	2
France	31	16				47	2
Iceland			30	30		60	2
Italy	23	30		30		83	3
Japan	29					29	1
Korea	30	29				59	2
The Netherlands	29			16		45	2
Norway	30		10			40	2
Spain			28			28	1
United States	16	97	28			141	3

All Countries							5
- Participants (n)	246	220	126	102	89	783	
- Participants (%)	31.4	28.1	16.1	13.0	11.4	100.0	
- Study sites	8	6	5	4	1	24	

### Data Collection

The five assessment instruments used in this study were the interRAI Long Term Care Facility (interRAI LTCF), interRAI Home Care (interRAI HC), interRAI Post Acute Care (interRAI PAC), interRAI Palliative Care (interRAI PC), and interRAI Mental Health (interRAI MH). All items are coded using the same assessment approach; namely, the assessor uses all sources of information and then exercises clinical judgement as to the most appropriate answer based on standardized coding guidelines provided in the instrument's training manual. Most items permit the use of multiple information sources including personal interviews, review of the chart, direct observation of the person, communication with informal caregivers, and use of clinical communication between health care staff (e.g., tracking forms, clinical correspondence). However, a limited number of items are restricted to recording only the person's self report (e.g., self-rated health; self-rated mood items dealing with depression, anxiety and anhedonia; personal goals of care). All items include standardized responses sets with item definitions, inclusions/exclusions, and observational time frames provided in the manual and on the assessment form. As noted earlier, all assessments include a set of common core data elements, which are the primary focus the present analyses, as well as specialized items unique to that service setting. The items typically are rated based on the presence or absence of a condition, frequency of its occurrence in a standardized time frame (typically three days), or severity based on anchor terms defined in the assessment manual (e.g., pain severity). The number of common and unique items for each instrument examined in the present study are reported in Table 2.

**Table 2:** Distribution of selected characteristics by interRAI instrument

**Variable**	**interRAI Assessment Instrument**					
	Long-Term Care Facility (LTCF)	Home Care (HC)	Palliative Care (PC)	Post-Acute Care (PAC)	Mental Health (MH)	All Instruments
Age group						
- <65	3.5	10.4	13.8	8.3	-^1^	9.9
- 65–84	50.9	60.4	46.8	68.1		57.5
- 85+	45.6	29.2	39.4	23.6		32.6
Female	72.3	62.1	58.7	66.0	-	65.7
Widowed	52.7	47.9	43.2	36.9	5.7	42.4
Impaired in decision making	83.4	45.7	65.9	38.2	43.8	59.5
Rarely/never understands others	4.9	1.5	18.0	0.0	-	5.5
Makes negative statements	23.6	17.4	16.5	10.8	17.9	18.5
Sad/pained facial expressions	22.4	12.5	22.9	27.4	47.2	23.5
Any aggressive behaviour^2^	17.3	0.5	-	-	25.8	12.4
Hallucinations or delusions	1.6	2.3	-	0.9	16.8	3.8
Early Loss ADL: Personal Hygiene Impaired	65.8	32.9	77.8	30.1	19.1	48.5
Mid Loss ADL: Walking Impaired	57.7	38.2	78.6	42.7	7.9	48.1
Late Loss ADL: Eating Impaired	21.8	18.3	57.6	4.8	7.9	22.7
Pain not present	61.9	34.2	42.4	41.6	0.0	41.3
Severe/excruciating pain present	6.9	23.7	24.8	8.9	-	15.8
Non-smoker	93.1	90.4	93.5	95.1	57.3	92.6
Falls	10.8	15.7	9.4	30.3	0.0	13.2
Hospitalized in last 90 days^3^	12.2	20.3	-	-	-	15.9

N	246	220	126	102	89	783

In some cases, items are not available because they were not gathered as part of the study protocol or they are not available for specific instruments.

Any occurrence of verbal abuse, physical abuse, socially inappropriate behaviour, or resisting care

Excludes psychiatric admissions

In each participating site, two health professionals completed their assessment of the same individual independently at different times. Assessors were blinded to others' results and they were not permitted to discuss the case with each other, nor were they permitted to exchange information; however, they were both able to access the persons chart when completing their assessment. The intent of this approach was to use a conservative methodology for evaluating inter-rater reliability in a manner that would mimic real-world assessment experiences. This methodology provides a more realistic appraisal of inter-rater reliability than would be obtained from use of artificial case examples or simultaneously completed assessments.

It was not the purpose of the study to make comparisons between sites or countries. Therefore, the protocol did not call for selection of randomized samples, and there was no requirement for countries to target common populations or settings. In order to reduce burden on staff and to simplify the approach for obtaining consent, convenience samples were used, but study coordinators were encouraged to obtain data for heterogeneous samples in order to test the applicability of the instruments in diverse clinical populations.

The study protocol required dual assessments to be done within 72 hours, but staff were encouraged to complete them in less time particularly in settings with a higher risk of rapid rate of clinical change (e.g., post-acute or palliative care). The actual number of hours between assessments was not recorded electronically, so it is not possible to estimate the average time between assessments.

Assessors were trained to use a variety of information sources, such as direct observation, interviews with the person under care, family, friends, or formal service providers, and review clinical records, both medical and nursing. The assessors were ordinary clinical staff, external research staff, or a mixture of both. Most assessors were nurses, but other professionals were also used. In line with interRAI's standard approach to coding, they were all instructed to exercise their best clinical judgment in order to record observations based on their evaluation of the most accurate information source.

All assessments were recorded in paper form and either entered locally into a database or sent to the project team for transcribing and analysis. Data from all countries were then combined into a single analytic data set for the cross national analyses.

Assessors were asked to track the time used to complete the assessment and to fill out a debriefing form on their experience in doing the assessment. This information was used by local project coordinators in participating countries to monitor how close the instruments were to meeting the target completion time and to collect comments that could be useful in future discussions regarding the refinement of the instruments. However, since the forms were considered an optional part of the protocol, this information was not always forwarded to the project coordinators and these data were not compiled electronically for further analysis. That said, there were no international reports of major problems with the study protocol or draft versions of instruments reported to the study team.

### Analysis

The reliability of the various interRAI instruments was evaluated mainly with weighted kappa coefficients using Fleiss-Cohen weights [24]. For binary items, ordinary kappa coefficients were used. According to Landis and Koch[25], kappa values below 0.40 should be considered poor, between 0.41 to 0.60 should be considered moderate, 0.61 to 0.80 should be considered substantial, and above 0.81 should be considered almost perfect. However, the stability of kappa estimates is affected by the distributional properties of items. For example, binary items with less than five percent of cases in one of the two values will yield unstable estimates, even with sample sizes of several hundred cases.

Kappa values are generally preferred as indicator of reliability over percentage agreement, because the latter may under-represent the reliability of multi-level items with modest disagreements regarding severity of a given problem. Percentage agreement will also over-represent reliability for binary items with highly skewed distributions.

### Ethics

Ethical considerations were addressed by the appropriate research ethics committee in each country providing data in accordance with national guidelines.

## Results

Table 2 provides a summary of sample characteristics for the five sectors included in the present study. Not surprisingly, there was considerable cross-sector variability in the types of persons being assessed. The residents of long term care facilities tended to be older, were more likely to be female, and had substantial higher rates of cognitive and ADL impairment than participants from other sectors. Pain rates were highest in palliative care settings, although pain was also very prevalent in the home care sample. The mental health sample had higher rates of depression and behaviour disturbance than was evident in other populations. These sample differences, though not surprising, are informative because they confirm the relatively distinctive natures of the populations considered here. On the other hand, the results also point to the fact that most of the issues considered in the interRAI suite are relevant to at least a subset of persons receiving care in most service settings.

**Figure 1:**
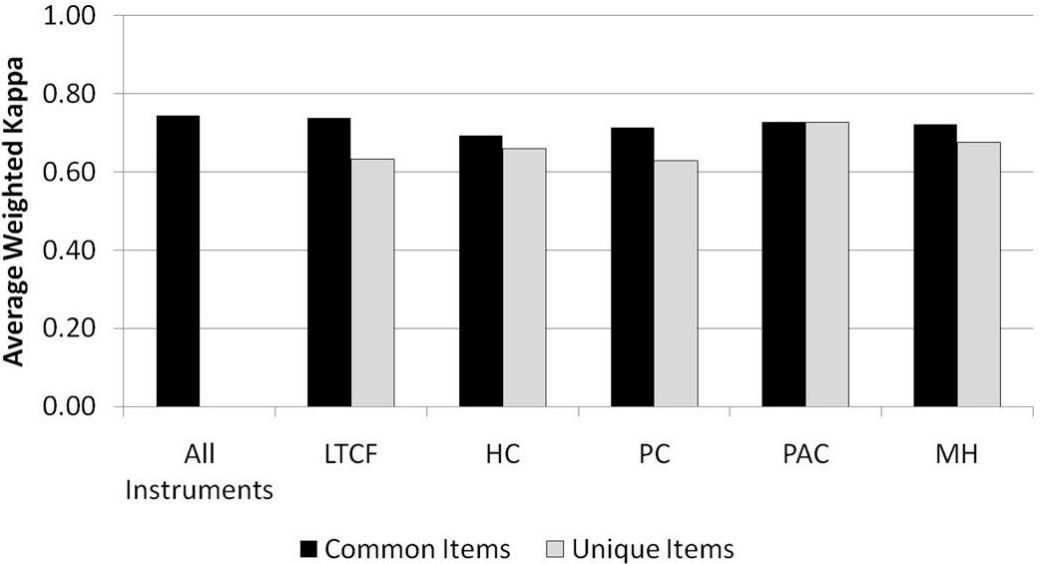
**Average weighted kappa value by interRAI instrument and type of item**.

Figure 1 provides the kappa values (binary items) and weighted kappa values for the total sample, including all tested instruments. For every instrument, kappa or weighted kappa values for items that also appear in at least one of the other instruments are presented separately from values of items that appear in only that instrument. The overall kappa mean value of all 161 items, common to two or more instruments, was 0.75. When considering the core items that appear in multiple instruments, the LTCF had the highest mean kappa (0.74) and the HC instrument the lowest (0.69). That said, all instruments can be considered to have substantial overall reliability based on Landis and Koch's [25] convention for interpreting kappa values.

The number of specialized items that were unique to individual instruments varied from as few as 8 items in the PAC to as many as 170 items in the MH. Considering these specialized items, the PAC showed the highest mean kappa value (0.73), while the other instruments varied between 0.63 and 0.68 (see Figure 1). These results are particularly encouraging because the specialized items tend to be among the newer items in the interRAI suite, whereas the core items are usually refinements of items that have been available for 10 or more years.

Table 3 shows examples of the mean kappa values for various assessment sections considering both the overall performance in the total sample and the means from each instrument tested. The items with the best mean kappa values in the total sample were Activities of Daily Living, discharge potential, therapies prescribed or given, height and weight, understanding others, IADL performance, standing, and incontinence. All of these items had a mean (weighted) kappa of 0.80 or better. The lowest mean kappa values were obtained for items related to vision, hearing, nutrition, delirium, some mood items, skin condition, health instability, dental health, hospital use, and social contacts. Nonetheless, these items had moderate to substantial agreement with the large majority obtaining kappa values between 0.60 and 0.70. For example, four items on delirium yielded 19 kappa values across five instruments (one of the delirium items is not used in the mental health instrument). These items had kappa values ranging from 0.52 for disorganized speech in the interRAI HC to 0.82 for mental function varies over the course of the day in the interRAI PAC. Sixteen of the 19 kappa values for delirium items were above 0.60.

**Table 3:** Average weighted kappa values by type of item and interRAI instrument

Item Domains	Number of Items	Long term care facility (LTCF)	Home Care (HC)	Palliative Care (PC)	Post Acute Care (PAC)	Mental Health (MH)	All Instruments
		148 common 48 unique	112 common 62 unique	63 common 64 unique	120 common 8 unique	48 common 170 unique	Weighed Kappa^4^
Sex	1	0.96	0.93	0.98	0.98		0.96
Marital Status	1	0.93	0.92	0.90	0.94	0.92	0.93
ADL	9	0.87	0.90	0.81	0.84	0.92	0.89
Discharge status	2	0.65	-	-	0.39	0.86	0.87
Height, weight	2	0.82	0.85	-	0.96	0.81	0.84
Stage of pressure ulcer	1	0.85	0.87	0.80	0.82	-	0.84
Communication	2	0.76	0.75	0.88	0.80	0.82	0.83
Tobacco, Alcohol	2	0.91	0.73	0.78	0.71	0.92	0.82
Allergy to drugs	1	0.91	0.74	0.88	0.76	-	0.82
IADL performance	6	-	0.77	-	0.74	-	0.82
IADL capacity	4	-	0.79	-	0.74	-	0.78
Standing	2	0.83	NA	-	0.75	-	0.81
Continence	3	0.90	0.76	0.72	0.63	0.91	0.80
Treatments	8	0.83	0.93	0.79	0.76	-	0.80
Thoughts	3	0.73	0.75	-	0.41	0.66	0.79
Cognition	4	0.77	0.65	0.83	0.69	0.87	0.79
Falls, fractures	3	0.86	0.65	0.61	0.82	0.73	0.77
Prevention	4	0.65	0.77	-	-	-	0.74
Pain	4	0.63	0.76	0.69	0.73	0.71	0.73
Legal status	5	0.73	0.61	-	0.76	0.94	0.73
Advance directives	4	0.63	-	0.87	0.83	1.00	0.73

For variable sets with a single item the kappa value reflects the value for that item alone. All other rows reflect the average kappa value for the types of items available for that domain area.

When considering individual items in specific instruments there was some cross-sector variability in kappa values. For example, the LTCF showed somewhat lower kappa values for discharge potential, pain, and skin. On the other hand, thirteen of the sections showed better kappa means than the total sample. Similar variability was evident for the other four instruments. However, it is striking that the large majority of items performed well in each of the five individual instruments. For only four items there were kappa values below 0.40 for one or more of these instruments.

**Figure 2:**
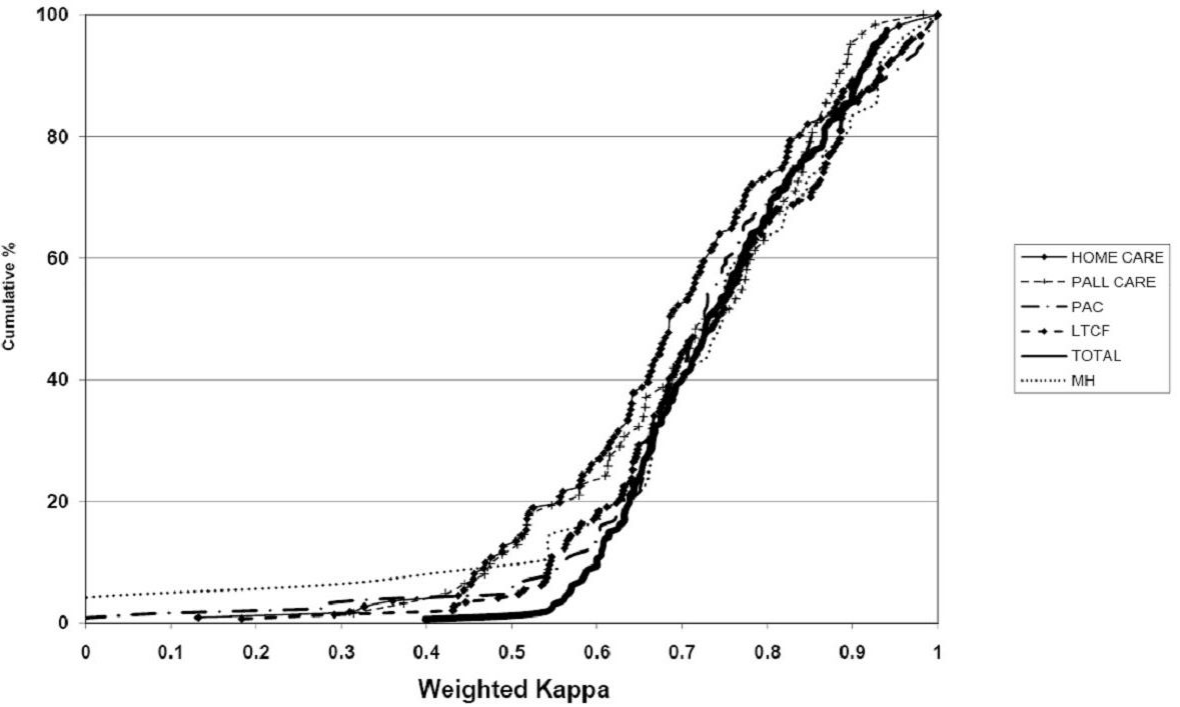
**Cumulative percentage of weighted kappa values by interRAI instrument**.

Figure 2 provides a cumulative percentage plot of kappa values by instrument to illustrate the consistency of item performance across the interRAI suite of instruments. The curves for all instruments combined and for individual instruments show almost identical patterns of reliability. When the results for all instruments pooled together, less than 10% of the items had a kappa value of 0.60 and only one value fell below 0.40 (0.399). Sixty percent of the values were above 0.70. However, when instruments are considered separately, the number of individual kappas below 0.40 is somewhat greater, ranging from a low of 3 items in the interRAI MH to a high of 17 items in the interRAI PAC.

The weakest curve was evident for home care clients, but even there 47% of the kappa values were above 0.70 and only four items had a kappa value below .40. The other instruments all had better distributions of the kappa values, the second worst being the palliative care, the best being the LTCF and the PAC.

## Discussion

Considerable clinical and scientific effort has been expended to establish the utility of earlier versions of the interRAI instruments for specific health care sectors. The present study is an important step forward because it provides strong evidence to demonstrate the reliability of the refined versions of these instruments comprising an integrated health information system for use in multiple care settings serving multiple populations. This new suite of instruments provides a scientifically sound information base to support cross-sectoral clinical and administrative decision-making. Although this is not a singular solution to the need for an integrated care system, it addresses at least one necessary condition for electronic health records that support health system integration: a common, reliable clinical language that can be used by interdisciplinary teams in diverse care settings.

There have been some efforts to create singular instruments that may be used in multiple post-acute care settings such as home care, rehabilitation hospitals and nursing homes (e.g., the Centers for Medicare & Medicare Services (CMS) CARE project). However, the interRAI suite of instruments differ from these tools in several important ways: a) applicability to a much broader range of service settings; b) use of a common core set of items linking all instruments combined with instrument-specific specialized items that are unique to the special needs of the populations served within specific care settings; and c) all instruments in the suite support multiple clinical and management applications for multiple audiences, including care planning, case mix classification, outcome measurement, quality management, and patient safety assessment.

The present results on reliability are compelling. Virtually all of the items tested met or exceeded standard cut-offs for acceptable reliability and a substantial proportion of items showed excellent reliability. This is particularly important given that a relatively conservative study protocol was used for completion of the assessments in order to provide a parallel approach to real-world assessment practices. These results demonstrate that motivated, appropriately trained staff can obtain high quality assessment results as part of normal clinical practice, and the domain areas assessed in the interRAI instruments can be assessed effectively in multiple settings serving persons of different ages with different strengths, preferences, and needs.

The development of any new assessment requires a substantial commitment of time and resources. The development of the new interRAI suite occurred over a five-year period of intensive research involving multidisciplinary teams of researchers based in 12 countries. An important consideration in international dissemination is translation and evaluation of cross-cultural applicability. Hence, it is important to establish not only the psychometric properties of assessment instruments in a single language. The present study demonstrates that the interRAI instruments are reliable in multiple languages and multiple countries. In addition, the interRAI development effort relies on extensive feedback from clinicians to ensure cultural appropriateness, clinical relevance and applicability of assessment items. The strategy of using all sources of information available to the assessor and the use of standardized coding rules and item definitions for interpretation by assessors provides the appropriate level of flexibility needed to ask questions in a cultural relevant manner while at the same time ensure international data standards. This is demonstrated in the present study by the high reliabilities achieved in North American, European, Asian, and Australian samples. interRAI is currently in the midst of ongoing translation (and back translation) of its entire suite of instruments into all of the languages represented in this, at present, 29-country network.

It was not surprising to note some modest cross-sector differences in reliability for the different interRAI instruments. While every instrument achieved high reliabilities overall and at the item level, the interRAI HC and interRAI PC obtained somewhat lower reliabilities than the other instruments in this study. Only about 15 percent of the items in the LTCF, PAC, and MH instruments had kappas below 0.60, whereas the HC and PC had about 25 percent of items below this value. There are a number of explanations for these findings. In the case of palliative care clients, the rate of clinical change and the fluctuations of symptoms and function at the end of life would be an important consideration for any protocol requiring repeat assessments at different time points. In the home care environment, staff members are more reliant on other, non-clinical informants (e.g., family members) to report on their observations over the previous three days. In facility settings, like nursing homes, psychiatric hospitals/units, or post-acute care, staff members are available on a 24-hour basis and use daily charting systems to monitor the person's status. Similarly, these facilities are more likely to have service utilization records (e.g., hospitalization) readily available in the chart accessible to staff at the time of the assessment, whereas home care case managers may need to rely on family members for this information. Therefore, it is not surprising to see a somewhat higher level of reliability in facility-based care settings.

The capacity to obtain reliable, compatible, clinically relevant data from multiple service settings has important implications for integrated systems of care. For example, if a person in one service setting has been assessed immediately prior to a transition to another setting (e.g., moving from inpatient psychiatry to community mental health; admission to a long term care facility from home care) staff may use the assessment performed in the prior service setting as baseline information to use in transitional care planning. The ability to utilize assessments done in other sectors should result in a net reduction of assessment burden in the overall system since the basis for reassessment should become significant clinical change rather than mistrust of data obtained from other health professionals. In addition, the earlier assessment provides baseline information that may be used to track clinical outcomes during the transition period (e.g., detection of delirium based on changes in assessment values between sectors). At the organizational level, the ability to link data from multiple service settings provides the opportunity to examine quality of care from the system-level rather than the organization level alone. For example, by linking interRAI MH discharge data with subsequent interRAI Community Mental Health data one would have the opportunity to benchmark mental health quality indicators related to clinical outcomes over the inpatient episode ***and ***over time as the person returns to living in the community.

While the present study yielded encouraging results related to reliability, there is considerable additional research underway related to the new interRAI suite of instruments. For example, the validity of items and scales in these instruments will be reported in future publications. The 12-country study described here included the additional simultaneous measurement of several other well-known scales (e.g., Mini-Mental State Examination). These data will be used to evaluate the concurrent validity of existing interRAI scales (e.g., Cognitive Performance Scale[26], Activities of Daily Living scales [27]) and to identify opportunities to develop new scales or refine existing scales by taking advantage of new interRAI items and extended response sets not available in previous versions. Another major research effort underway is the refinement of Clinical Assessment Protocols (CAPs) used as a basis for care planning with all instruments in the suite[28]. The new CAPs for the HC, LTCF, PAC, and AL instruments were completed in 2007, and reports on that research are in development. The CAPs for all remaining instruments should be completed by 2009.

Additional work is required to link the clinical data elements of the interRAI instruments to electronic health record standards such as the SNOMED-CT system [29]. For example, many of the items included in the MDS (predecessor to the interRAI LTCF) do not have equivalent terms in SNOMED-CT [30]. While the present research makes an important contribution by establishing uniform data standards across multiple interRAI instruments, the work to link these instruments to terminology systems in medical records has not been completed.

The international scope of the study resulted in some unavoidable study limitations. For example, although every effort was made to standardize the study methods, there may have been some country-level differences training approaches, staff skill levels, and data collection methods that were not detected here. The variations in which instruments were used across countries made it difficult to evaluate country-specific results for individual instruments. Similarly, it was not possible to evaluate the performance of all instruments within a specific country, since no single country evaluated more than three instruments. In addition, this study examined the level of agreement between two assessors for different instruments, but there was no requirement for either assessor to be considered a "gold standard" assessor.

An important strength of this study is the use of a methodology that produces conservative estimates of reliability. Assessors were directed to complete their assessments entirely independently at different times and without any communication between them. This approach is consistent with how assessments are done in actual clinical practice, since there are typically not sufficient resources available for multiple clinicians to complete assessments together. Reliability studies that allow dual assessments to be completed at the same time will obviously result in very high levels of agreement between assessors, but such methods bear little resemblance to how these instruments will be used in normal clinical practice.

## Conclusion

Producing international instruments, making them reliable, and confirming their validity is an important task for the interRAI research network. Very few similar endeavours have been reported in the literature, except for diagnostic standards such as the World Health Organization's International Classification of Diseases or the Diagnostic and Statistical Manual (DSM) for psychiatric conditions. Hence, the new suite of interRAI instruments can be considered to be an important step forward in achieving international standards for assessment of vulnerable populations with complex care and support needs.

The inter-rater reliability results from the early use of the new suite of interRAI instruments shows that most of the items in these instruments work very well in multiple service settings. This means that clinicians, administrators, policy makers, and researchers can have considerable confidence in the data obtained for different populations receiving services in different sectors of the health care system.

The opportunities for cross-sector and cross-national comparisons based on the interRAI instruments hold considerable promise for developing effective strategies for responding to major global challenges related to population aging and the expansion of disability. Having established a common metric to measure major questions of interest to service providers, planners, and policy makers, the capacity to use evidence to inform decision making at all levels of the health care system has increased greatly.

## Competing interests

The authors declare that they have no competing interests.

## Authors' contributions

JPH and GL jointly drafted the first version of the manuscript. JNM provided oversight to the international data collection, dataset preparation, and analysis for this manuscript. JPH, DF and other interRAI Fellows mentioned in the acknowledgements managed data collection in their respective countries. All other co-authors contributed toward conceptualization and revision of the manuscript, and they approved the final version.

## Pre-publication history

The pre-publication history for this paper can be accessed here:



## References

[B1] Jonsson PV, Finne-Soveri H, Jensdottir AB, Ljunggren G, Bucht G, Grue EV, Noro A, Bjornson J, Jonsen E, Schroll M (2006). Co-morbidity and functional limitation in older patients underreported in medical records in Nordic Acute Care Hospitals when compared with the MDS-AC instrument [1]. Age and Ageing.

[B2] Hirdes JP, Fries BE, Morris JN, Steel K, Mor V, Frijters D, LaBine S, Schalm C, Stones MJ, Teare G, Smith T, Marhaba M, Perez E, Jonsson P (1999). Integrated health information systems based on the RAI/MDS series of instruments. Healthcare management forum.

[B3] Carpenter GI (2006). Accuracy, validity and reliability in assessment and in evaluation of services for older people: The role of the interRAI MDS assessment system. Age and Ageing.

[B4] Morris JN, Hawes C, Fries BE, Phillips CD, Mor V, Katz S, Murphy K, Drugovich ML, Friedlob AS (1990). Designing the national resident assessment instrument for nursing homes. Gerontologist.

[B5] Fries BE, Schroll M, Hawes C, Gilgen R, Jonsson PV, Park P (1997). Approaching cross-national comparisons of nursing home residents. Age Ageing.

[B6] Morris JN, Fries BE, Steel K, Ikegami N, Bernabei R, Carpenter GI, Gilgen R, Hirdes JP, Topinkova E (1997). Comprehensive clinical assessment in community setting: applicability of the MDS-HC. J Am Geriatr Soc.

[B7] Landi F, Tua E, Onder G, Carrara B, Sgadari A, Rinaldi C, Gambassi G, Lattanzio F, Bernabei R (2000). Minimum data set for home care: A valid instrument to assess frail older people living in the community. Medical Care.

[B8] Carpenter I, Gambassi G, Topinkova E, Schroll M, Finne-Soveri H, Henrard JC, Garms-Homolova V, Jonsson P, Frijters D, Ljunggren G, Sørbye LW, Wagner C, Onder G, Pedone C, Bernabei R (2004). Community care in Europe. The Aged in Home Care project (AdHOC). Aging Clin Exp Res.

[B9] Hirdes JP, Marhaba M, Smith TF, Clyburn L, Mitchell L, Lemick RA, Telegdi NC, Perez E, Prendergast P, Rabinowitz T, Yamauchi K (2000). Development of the Resident Assessment Instrument – Mental Health (RAI-MH). Hospital quarterly.

[B10] Hirdes JP, Smith TF, Rabinowitz T, Yamauchi K, Perez E, Telegdi NC, Prendergast P, Morris JN, Ikegami N, Phillips CD, Fries BE (2002). The Resident Assessment Instrument-Mental Health (RAI-MH): Inter-Rater Reliability and Convergent Validity. Journal of Behavioral Health Services and Research.

[B11] Carpenter GI, Teare GF, Steel K, Berg K, Murphy K, Bjornson J, Jonsson PV, Hirdes JP (2001). A new assessment for elders admitted to acute care: Reliability of the MDS-AC. Aging (Milano).

[B12] Jonsson PV, Finne-Soveri H, Jensdottir AB, Ljunggren G, Bucht G, Grue EV, Noro A, Bjornson J, Jonsen E, Schroll M (2006). Co-morbidity and functional limitation in older patients underreported in medical records in Nordic Acute Care Hospitals when compared with the MDS-AC instrument [1]. Age and Ageing.

[B13] Gindin J, Walter-Ginzburg A, Geitzen M, Epstein S, Levi S, Landi F, Bernabei R (2007). Predictors of Rehabilitation Outcomes: A Comparison of Israeli and Italian Geriatric Post-Acute Care (PAC) Facilities Using the Minimum Data Set (MDS). Journal of the American Medical Directors Association.

[B14] Steel K, Ljunggren G, Topinkova E, Morris JN, Vitale C, Parzuchowski J, Nonemaker S, Frijters DH, Rabinowitz T, Murphy KM, Ribbe MW, Fries BE (2003). The RAI-PC: An assessment instrument for palliative care in all settings. American Journal of Hospice and Palliative Medicine.

[B15] Hawes C, Morris JN, Phillips CD, Mor V, Fries BE, Nonemaker S (1995). Reliability estimates for the minimum data set for nursing home resident assessment and care screening (MDS). Gerontologist.

[B16] Morris JN, Nonemaker S, Murphy K, Hawes C, Fries BE, Mor V, Phillips C (1997). A commitment to change: revision of HCFA's RAI. J Am Geriatr Soc.

[B17] Fries BE, Simon SE, Morris JN, Flodstrom C, Bookstein FL (2001). Pain in U.S. nursing homes: Validating a pain scale for the Minimum Data Set. Gerontologist.

[B18] Koehler M, Rabinowitz T, Hirdes J, Stones M, Carpenter GI, Fries BE, Morris JN, Jones RN (2005). Measuring depression in nursing home residents with the MDS and GDS: An observational psychometric study. BMC Geriatrics.

[B19] Mor V, Angelelli J, Jones R, Roy J, Moore T, Morris J (2003). Inter-rater reliability of nursing home quality indicators in the U.S. BMC Health Services Research.

[B20] Morris JN, Jones RN, Fries BE, Hirdes JP (2004). Convergent validity of minimum data set-based performance quality indicators in postacute care settings. American Journal of Medical Quality.

[B21] Hawes C, Fries BE, James ML, Guihan M (2007). Prospects and pitfalls: Use of the RAI-HC assessment by the Department of Veterans Affairs for home care clients. Gerontologist.

[B22] Gray LC, Bernabei R, Berg K, Finne-Soveri H, Fries BE, Hirdes JP, Jonsson PV, Morris JN, Steel K, Arino-Blasco S (2008). Standardizing Assessment of Elderly People in Acute Care: The interRAI Acute Care Instrument. J Am Geriatr Soc.

[B23] Martin LR, Hirdes JP, Fries BE, Smith TF (2007). Development and psychometric properties of an assessment for persons with ID-the interRAI ID. Journal of Policy and Practice in Intellectual Disabilities.

[B24] Fleiss JL (1981). Statistical methods for rates and proportions.

[B25] Landis JR, Koch GG (1977). The measurement of observer agreement for categorical data. Biometrics.

[B26] Morris JN, Fries BE, Mehr DR, Hawes C, Phillips C, Mor V, Lipsitz LA (1994). MDS Cognitive Performance Scale. J Gerontol.

[B27] Morris JN, Fries BE, Morris SA (1999). Scaling ADLs within the MDS. J Gerontol A Biol Sci Med Sci.

[B28] Fries BE, Morris JN, Bernabei R, Finne-Soveri H, Hirdes J (2007). Rethinking the Resident Assessment Protocols. J Am Geriatr Soc.

[B29] Kuziemsky CE, Lau F (2008). A comparative analysis of computer based hospice palliative care datasets in Canada. BMC Pall Care.

[B30] Harris MR, Chute CG, Harvell J, White A, Moore T (2003). Toward a National Health Information Infrastructure: A Key Strategy for Improving Quality in Long Term Care. Office of the Assistant Secretary of Planning and Evaluation.

